# The Development and Validation of a CT-Based Radiomics Nomogram to Preoperatively Predict Lymph Node Metastasis in High-Grade Serous Ovarian Cancer

**DOI:** 10.3389/fonc.2021.711648

**Published:** 2021-08-31

**Authors:** Hui-zhu Chen, Xin-rong Wang, Fu-min Zhao, Xi-jian Chen, Xue-sheng Li, Gang Ning, Ying-kun Guo

**Affiliations:** ^1^Department of Radiology, Key Laboratory of Birth Defects and Related Diseases of Women and Children of Ministry of Education, West China Second University Hospital, Sichuan University, Chengdu, China; ^2^PET/MR Department, GE Healthcare, Shanghai, China

**Keywords:** high-grade serous ovarian cancer, computed tomography, radiomics, lymph node, metastasis

## Abstract

**Purpose:**

To develop and validate a radiomics model for predicting preoperative lymph node (LN) metastasis in high-grade serous ovarian cancer (HGSOC).

**Materials and Methods:**

From May 2008 to January 2018, a total of 256 eligible HGSOC patients who underwent tumor resection and LN dissection were divided into a training cohort (n=179) and a test cohort (n=77) in a 7:3 ratio. A Radiomics Model was developed based on a training cohort of 179 patients. A radiomics signature (defined as the Radscore) was selected by using the random forest method. Logistics regression was used as the classifier for modeling. An Integrated Model that incorporated the Radscore and CT_reported LN status (CT_LN_report) was developed and presented as a radiomics nomogram. Its performance was determined by the area under the curve (AUC), calibration, and decision curve. The radiomics nomogram was internally tested in an independent test cohort (n=77) and a CT-LN-report negative subgroup (n=179) using the formula derived from the training cohort.

**Results:**

The AUC value of the CT_LN_report was 0.688 (95% CI: 0.626, 0.759) in the training cohort and 0.717 (95% CI: 0.630, 0.804) in the test cohort. The Radiomics Model yielded an AUC of 0.767 (95% CI: 0.696, 0.837) in the training cohort and 0.753 (95% CI: 0.640, 0.866) in the test. The radiomics nomogram demonstrated favorable calibration and discrimination in the training cohort (AUC=0.821), test cohort (AUC=0.843), and CT-LN-report negative subgroup (AUC=0.82), outperforming the Radiomics Model and CT_LN_report alone.

**Conclusions:**

The radiomics nomogram derived from portal phase CT images performed well in predicting LN metastasis in HGSOC and could be recommended as a new, convenient, and non-invasive method to aid in clinical decision-making.

## Introduction

Epithelial ovarian cancer (EOC) has the highest mortality rate among all gynecological malignancies, and approximately two-thirds of women are staged as International Federation of Gynecology and Obstetrics (FIGO) III–IV (1). High-grade serous ovarian cancer (HGSOC) accounts for 70% of EOC patients and is the lethal histological subtype (2–4). Lymph node (LN) metastasis in HGSOC patients is observed in up to 75% of patients with stage III–IV disease and in 25% of patients with stage I–II disease (5, 6). LN status has an important impact on the FIGO stage of EOC (7–11). For example, stage I patients with LN metastasis will be rediagnosed as stage III or IV (12–17). Moreover, LN metastasis in different sites for ovarian cancer may have not the same impact on progression-free survival (PFS) and overall survival (OS) (11, 18–20). In the study by Gallotta et al. (19), the patients with metastatic hepatoceliac lymph nodes (HCLNs) experienced worse PFS than the patients with uninvolved ones, but clinicians often underestimate the true prevalence of disease in this area due to the lack of effective methods before surgery. Furthermore, patients with stage I disease (10) and those suitable for neoadjuvant chemotherapy (21) also should accurately assess the LN status preoperatively. However, the gold standard methods for EOC staging are surgery and histopathologic diagnosis, so it is necessary to explore non-invasive methods to predict LN metastatic preoperatively for aiding in clinical decision-making.

LN metastasis has been evaluated using computed tomography (CT), magnetic resonance imaging (MRI), and positron emission tomography with CT (PET-CT) to measure LN size (22). LN positive for metastasis is defined as larger than 10 mm in the short-axis diameter and central necrosis in the portal phase (23). Presently, CT is the first-line imaging method for ovarian cancer staging and follow-up according to the European Society of Urogenital Radiology and the American College of Radiology guidelines (22, 24). However, its accuracy in predicting LN metastasis is unsatisfactory, with a sensitivity of 48%-80% (25). MRI is recommended as a second-line technique, with a sensitivity of 54.7% in predicting LN metastasis (9, 25). PET-CT may improve staging accuracy, but it has a high rate of false-positive results (24). These low efficacies have led to a considerable proportion of patients being understaged or overstaged. Therefore, it is critical to evaluate the LN metastasis of ovarian cancer in a non-invasive way, which may guide optimal treatment planning and help to determine prognosis. However, it is difficult to achieve accurate preoperative LN staging with the currently available methods.

Radiomics is the process of the conversion of medical images into high-dimensional, mineable data *via* the high-throughput extraction of quantitative features, followed by subsequent data analysis for decision support. The radiomics approach could non-invasively provide rich information on diseases, such as assessing the diagnosis of diseases, evaluating prognosis, and predicting treatment response (26, 27). At present, radiomics is mainly applied in oncology to aid in improving clinical decision-making (28). Previous studies have suggested that the preoperative prediction of LN metastasis can be improved by using a radiomics-based approach for colorectal cancer, bladder cancer, intrahepatic cholangiocarcinoma, gastric cancer, and lung adenocarcinoma (23, 27, 29–32). To the best of our knowledge, there are few studies that have determined whether a radiomics signature would enable the superior prediction of LN metastasis in patients with HGSOC. We hypothesized that the radiomics signature might help to improve the diagnostic value of LN metastasis in HGSOC by combining traditional imaging features.

Therefore, in our study, we aimed to develop and validate a radiomics nomogram that incorporated both a radiomics signature and traditional imaging features for the individual preoperative prediction of LN metastasis in patients with HGSOC.

## Materials and Methods

This retrospective study was approved by the Institutional Review Board of West China Second University Hospital (No. 2020173), and we pledged to abide by the Declaration of Helsinki (2013 EDITION) in accordance with the relevant medical research rules of China in the study. The requirement for written informed consent was waived. All patient sensitive information was treated with strict secrecy and used solely for the purposes of this study.

### Patients

From May 2008 to January 2018, a total of 486 consecutive women with pathologically confirmed HGSOC who underwent appropriate surgical staging and/or debulking surgery with pelvic and/or para-aortic lymph node dissection were reviewed from our institutional database. The inclusion criteria were as follows: (1) pathologically confirmed HGSOC with definite LN stage, without neoadjuvant chemotherapy (NAC); (2) preoperative contrast-enhanced CT scans; (3) no chemotherapy or radiation therapy prior to CT scans; and (4) preoperative enhanced abdominal CT examination within 2 weeks prior to surgery.

The exclusion criteria were as follows: (1) lack of definite information on postoperative LN status (n=89); (2) received NAC or radiotherapy before surgery (n=73); (3) lack of contrast-enhanced CT scans at our institution (n=53); (4) any artifacts within the scan area that displayed the lesion (n=10); and (5) the scan area did not cover the entire lesion (n=5).

**Figure 1:**
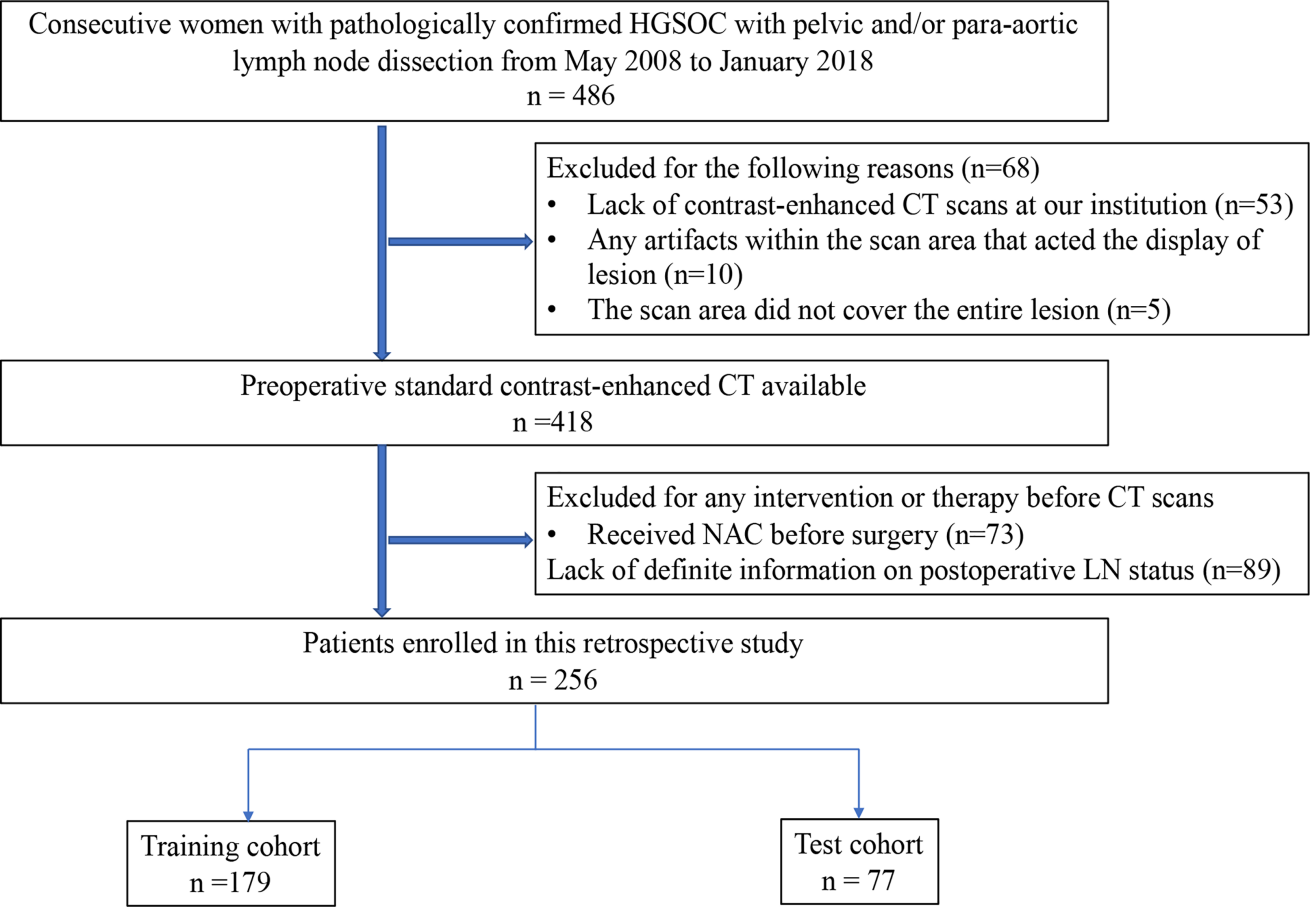
Recruitment pathway for the patients in this study.

A total of 256 patients who met the criteria were randomly divided into a training cohort (n=179) and a test cohort (n=77) in a 7:3 ratio (**Figure 1**).

**Figure 2:**
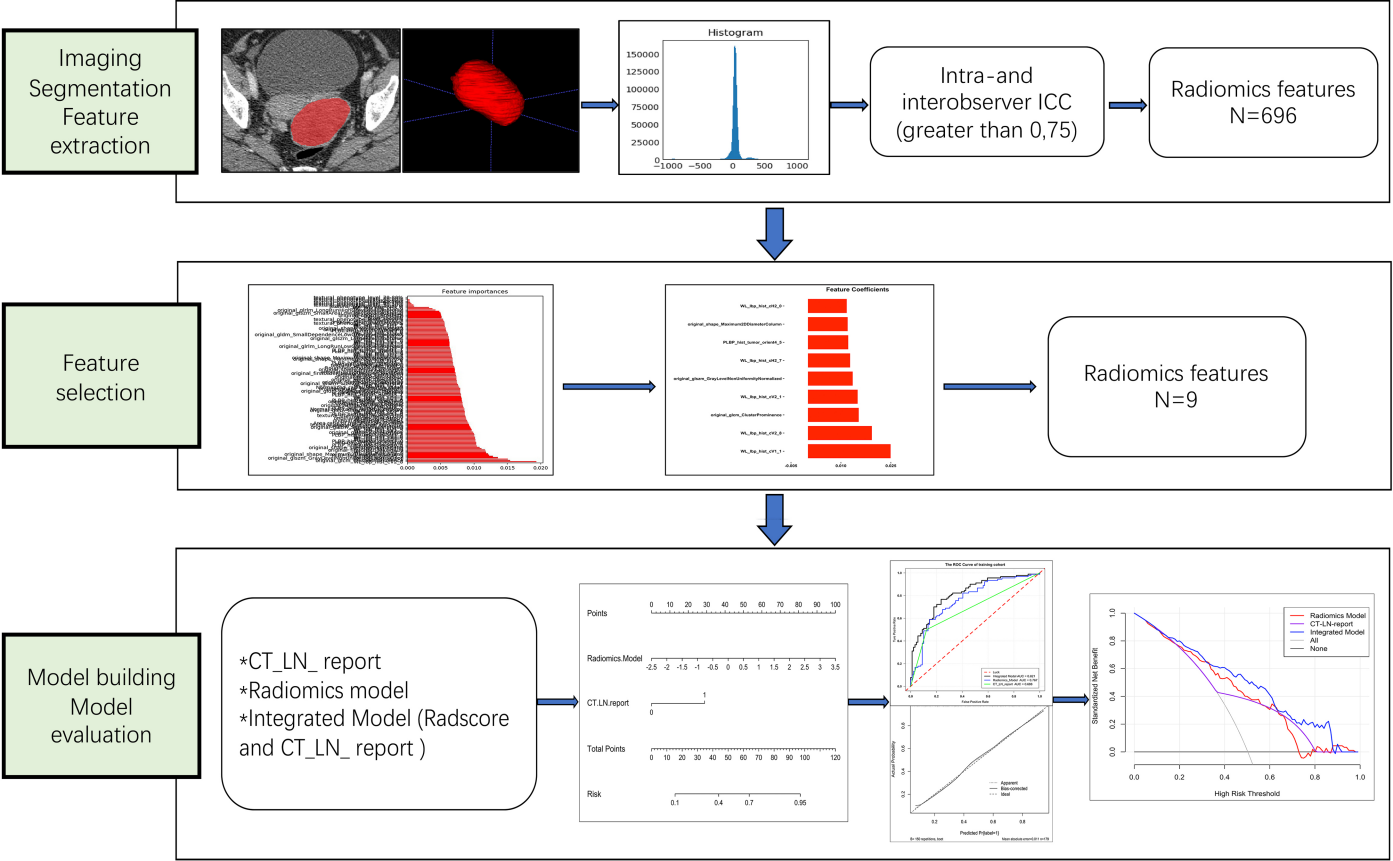
Workflow of the steps in our study. First, tumors were manually segmented, and radiomic features were extracted from within the defined tumor contours on CT images. Second, for feature selection, the random forest method was used to rank the remaining features in order of importance according to different kinds of permutations and combinations. Finally, the performance of the radiomics nomogram was developed based on the Radscore and CT_LN_report in the training cohort and assessed by ROC curve, calibration curve, and decision curve analyses.

Clinical data, including age, family history of cancer, preoperative carbohydrate antigen 125 (CA125) level, carbohydrate antigen 199 (CA199) level, and dates of baseline CT imaging, were obtained from medical records. Our study flow diagram is shown in **Figure 2**.

### CT Image Acquisition and Radiologic Evaluation

One multidetector CT scanner (Brilliance 6, Philips Medical System, Best, Netherlands) was performed on 113 patients. The scan area was from the pubic symphysis to the diaphragm, including the non-enhancement scan, arterial phase, and portal venous phase. The CT scan parameters were as follows: tube voltage= 120 kVp; tube current= 100–320 mA; beam pitch=0.9; reconstruction thickness= 2 mm; reconstruction interval= 1 mm; matrix= 512 × 512. Contrast medium (80–100 ml; Iohexol, GE healthcare, USA) was injected into the antecubital vein using a mechanical injector at a rate of 2.5–3.5 ml/s. The arterial phase and portal venous phase times were 20–25 s and 60–70 s after contrast agent injection, respectively.

Another multidetector CT scanner (NeuViz 128 1.0, China) was performed on 143 patients. The CT scan parameters were as follows: tube voltage= 120 kVp; tube current= automatic milliampere setting with a range of 100–500 mA; beam pitch <1; reconstruction thickness= 1 mm; reconstruction interval= 0.5 mm; matrix= 512 × 512. The method and process of the contrast-enhanced CT scan were the same as those of the multidetector CT scanner (Brilliance 6, Philips Medical System, Best, Netherlands).

All CT images were reviewed by two radiologists (reader 1 and reader 2, with 8 and 12 years of gynecological imaging experience, respectively) in consensus to evaluate the following traits: (a) tumor size (the maximum diameter on transverse images), (b) laterality (the number of tumors), (c) tumor stage, and the maximal short-axis diameter of the largest LN in the para-aorta or pelvic cavity were recorded. The maximal short-axis diameter (>10 mm) or central necrosis in the portal venous phase was defined as LN positive for metastasis (23, 33). The CT-reported LN status hereinafter referred to as CT-LN-report. The two radiologists were blinded to the pathologic details. Any disagreement was resolved by consultation.

### Tumor Segmentation

The preoperative CT images in all patients with HGSOC were downloaded by means of Digital Imaging and Communications in Medicine (DICOM) data from the picture archiving and communication system (PACS). All CT DICOM images were collected from two different scanners with different scanning parameters. Portal venous phase images at 1 or 2 mm thickness were chosen for radiomic feature extraction. The regions of interest (ROIs) covered the whole tumor and were manually delineated along the tumor contour on each transverse section by using open-source imaging software (ITK-SNAP, version 3.6.1; www.itksnap.org) for 3-D segmentation.

The intra- and interclass correlation coefficients (ICCs) were calculated to ensure reproducibility and accuracy. Initially, 50 patients with CT images were randomly chosen for ROI segmentation and radiomic feature extraction. Then, to assess interobserver reliability, ROI segmentation was performed in a blinded fashion by reader 1 (with 8 years of experience in gynecological imaging) and reader 2 (with 12 years of experience in gynecological imaging). To evaluate intraobserver reliability, reader 1 repeated the same procedure 1 month later. The remaining image segmentation was completed by reader 1. An ICC greater than 0.75 was considered to indicate good agreement of the feature extraction (34).

### Radiomics Feature Extraction, Normalization, and Signature Construction

In total, 696 radiomics features were automatically extracted from each segmented ROI by Intelligence Foundry Version 3.0.3. A (GE Healthcare, USA). All features were calculated in three-dimensional directions within the whole tumor volume. To minimize the different kinds of CT parameter variations, we normalized the imaging parameters using Z-score standardization. The formula was as follows: z =(x-μ)/σ, where *x* refers to the original value, μ refers to the mean value, and σ refers to the standard deviation.

The Spearman correlation coefficient was calculated, and 0.95 was used as the threshold to remove highly correlated features. Then, the importance value of the remaining CT image features was calculated by means of Random Forest algorithm, and we selected optimal features in accordance with feature. Logistics regression was used as the classifier for modeling, with penalty parameter tuning conducted by five-fold cross-validation. A radiomics score (Radscore) was calculated for each patient *via* a linear combination of selected features that were weighted by their respective coefficients. The Radiomics Model was constructed based on Radscore.

### Development and Validation of the Integrated Model

An Integrated Model incorporating Radscore and CT_LN_report was developed and presented as a radiomics nomogram in the training cohort. The calibration of the radiomics nomogram was assessed with a calibration curve. The performance of the radiomics nomogram was then internally tested in an independent test cohort and the CT_LN_report negative sample.

### Clinical Use of the Radiomics Nomogram

The decision curve analysis (DCA) was used to estimate the clinical utility of the radiomics nomogram, Radscore, and CT_LN_report by calculating the net benefits for a range of threshold probabilities in the training and test cohorts.

### Statistical Tools

We performed the statistical analysis in R (version 3.6.4; http://www.Rproject.org) and IBM SPSS Statistics version 25.0. Common comparisons of patient characteristics were conducted by the two-sample t-test or Mann-Whitney U test for continuous variables. Fisher’s exact test or the χ2 test was used for categorical variables. The area under the curve (AUC) was calculated to predict the discrimination performance of the Radiomics Model, Integrated Model, and CT_LN_report in both the training and test cohorts. The DeLong non-parametric was used to compare AUCs among prediction models. Decision curve analysis was used to calculate the net benefit from the use of the Integrated Model, Radiomics Model, and CT_LN_report at different threshold probabilities in the training and test cohorts. A two-sided P value less than 0.05 was considered to indicate statistical significance.

## Results

### Patient Characteristics

All patient characteristics in the training and test cohorts are shown in **Table 1**. There were no differences in clinical and radiologic characteristics between them. The AUC value of the CT_LN_report was 0.688 (95% CI: 0.626, 0.759) in the training cohort and 0.717 (95% CI: 0.630, 0.804) in the test cohort, with sensitivities of 50 and 48.7%, respectively (**Table 2**). In total, 65 patients (50.4%; 65 of 129) with LN metastasis were understaged, and 13 patients (10.2%; 13 of 127) without LN metastasis were overstaged according to the pathologic examination for LN metastasis.

**Table 1:** Clinical characteristics of the patients in the training and test cohorts.

	Training Cohort (n = 179)			Test Cohort (n = 77)		
	Negative for LN Metastasis	Positive for LN Metastasis	P Value	Negative for LN Metastasis	Positive for LN Metastasis	P Value
No. Patients	89	90		38	39	
Age (y)	53.69 ± 8.35	50.32 ± 7.24	0.004	54.37 ± 7.69	48.31 ± 7.34	0.001
CA125 (miu/ml)			0.682			0.615
≧35 miu/ml	87 (97.8%)	86 (95.6%)		36 (94.7%)	38 (97.4%)	
<35 miu/ml	2 (2.2%)	4 (4.4%)		2 (5.3%)	1 (2.6%)	
CA199 (miu/ml)			0.072			0.481
≧30.9 miu/ml	7 (7.9%)	16 (17.8%)		5 (13.2%)	3 (7.7%)	
<30.9 miu/ml	82 (92.1%)	74 (82.2%)		33 (86.8%)	36 (92.3%)	
Ascites			0.074			0.335
Present	73 (82%)	82 (91.1%)		30 (78.9%)	34 (87.2%)	
Absent	16 (18%)	8 (8.9%)		8 (21.1%)	5 (12.8%)	
CT_T_stage			0.000			0.000
I–II	34 (38.2%)	7 (7.8%)		13 (34.2%)	0 (0%)	
III–IV	55 (61.8%)	83 (92.2%)		25 (65.8%)	39 (100%)	
Laterality (%)			0.069			0.014
Unilateral	34 (38.2%)	23 (25.6%)		19 (50%)	9 (23.1%)	
Bilateral	55 (61.8%)	67 (74.4%)		19 (50%)	30 (76.9%)	
CT_tumor size	85.23 ± 35.89	83.76 ± 28.71	0.762	86.78 ± 38.29	78.54 ± 27.59	0.281
CT_LN_report			0.000			0.000
Negative	78 (87.6%)	45 (50%)		36 (94.7%)	20 (51.3%)	
Positive	11 (12.4%)	45 (50%)		2 (5.3%)	19 (48.7%)	
Radscore	−0.424 ± 0.875	0.441 ± 0.905	0.000	−0.349 ± 1.529	0.514 ± 0.972	0.004

CA125, carbohydrate antigen 125; CA199, carbohydrate antigen 199; CT_T_stage, CT_reported tumor stage; CT_tumor size, CT_reported tumor size; CT_LN_report, CT_reported lymph node status.

**Table 2:** Diagnostic efficiency of the CT_LN_report, Radiomics Model, and Integrated Model in the training and test cohorts.

	Training Cohort			Test Cohort		
	Sen	Spe	AUC (95% CI)	Sen	Spe	AUC (95% CI)
CT_LN_report	0.500	0.876	0.688 (0.626–0.759)	0.487	0.947	0.717 (0.630–0.804)
Radiomics model	0.589	0.854	0.767 (0.696–0.837)	0.605	0.872	0.753 (0.640–0.866)
Integrated Model	0.767	0.764	0.821 (0.760–0.882)	0.846	0.789	0.843 (0.750–0.936)

Spe, Specificity; Sen, Sensitivity.

### Feature Selection and Radiomics Model Construction

**Figure 3:**
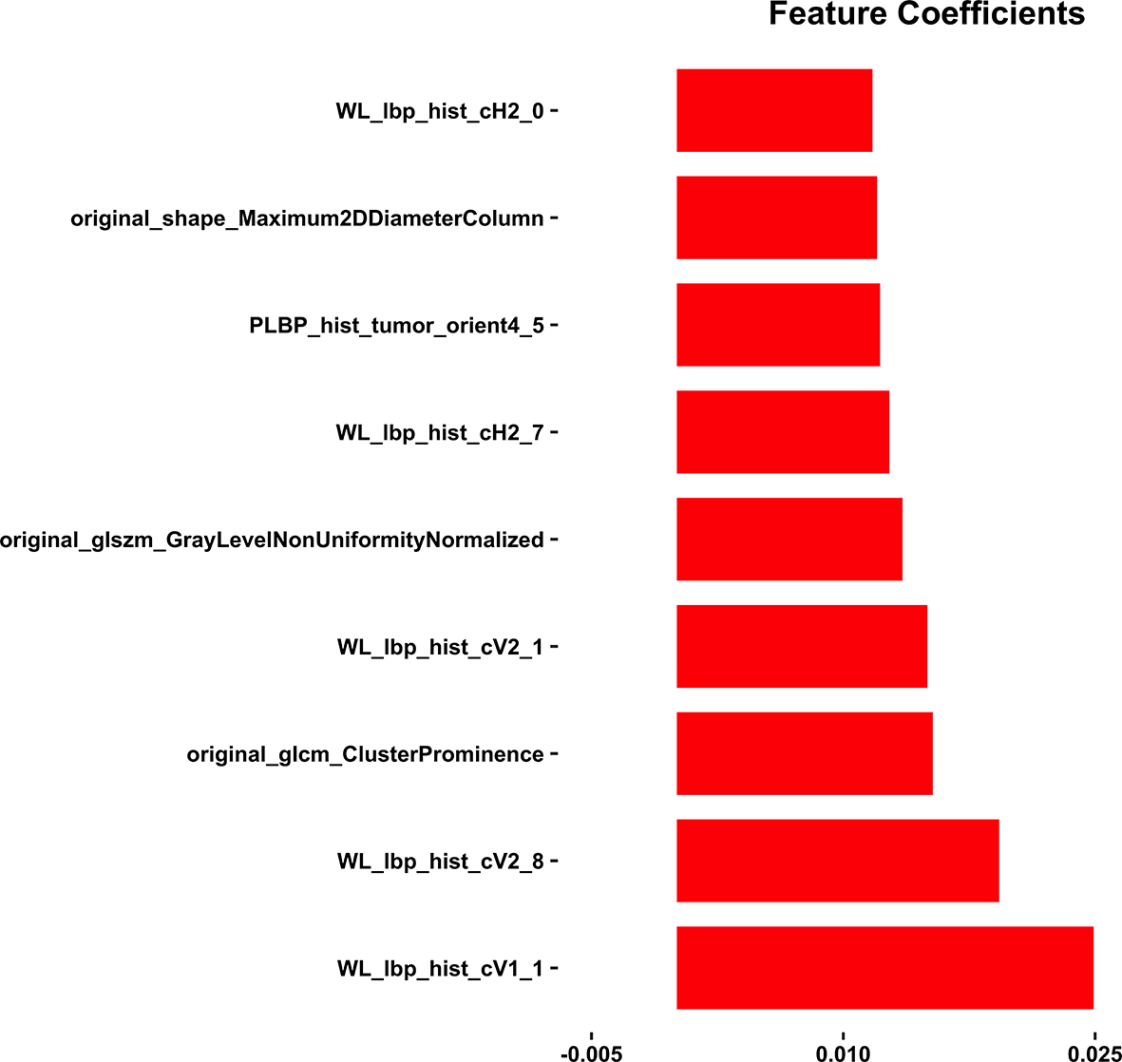
The coefficients of the nine radiomics features by using the random forest (RF) method.

Nine LN status-related features with non-zero coefficients in the random forest model were selected based on the training cohort (**Figure 3**). The Radscore was calculated by using the following formula:

$$\begin{array}{c} \text{Radiomics score}=0.025\times \text{WL}\_\text{lbp}\_\text{hist}\_\text{cV}1\_1\\ +\;0.019\times \text{WL}\_\text{lbp}\_\text{hist}\_\text{cV}2\_8\\ +\;0.015\times \text{original}\_\text{glcm}\_\text{ClusterProminence}\\ +\;0.015\times \text{WL}\_\text{lbp}\_\text{hist}\_\text{cV}2\_1+\;0.014\\ \times \text{original}\_\text{glszm}\_\text{GrayLevelNonUniformityNormalized}\\ +\;0.013\times \text{WL}\_\text{lbp}\_\text{hist}\_\text{cH}2\_7\\ +\;0.012\times \text{PLBP}\_\text{hist}\_\text{tumor}\_\text{orient}4\_5\\ +\;0.012\times \text{original}\_\text{shape}\_\text{Maximum}2\text{DDDiameterColumn}\\ +\;0.012\times \text{WL}\_\text{lbp}\_\text{hist}\_\text{cH}2\_0 \end{array}$$

The Radiomics Model was constructed based on Radscore, and the distributions of the Radscore for each patient in the training and test cohorts are shown in **Appendix E1** and **E2**.

### Diagnostic Validation of the Radiomics Model

There was a difference in the Radscore between patients with and without LN metastasis in the training cohort (0.441 ± 0.905 *vs* −0.424 ± 0.875; P= 0.000) and the test cohort (0.514 ± 0.972 *vs* −0.349 ± 1.529; P= 0.004). The Radiomics Model yielded an AUC of 0.767 (95% CI: 0.696, 0.837) in the training cohort and 0.753 (95% CI: 0.640, 0.866) in the test cohort (**Table 2**).

### Development, Performance, and Validation of the Integrated Model

**Figure 4:**
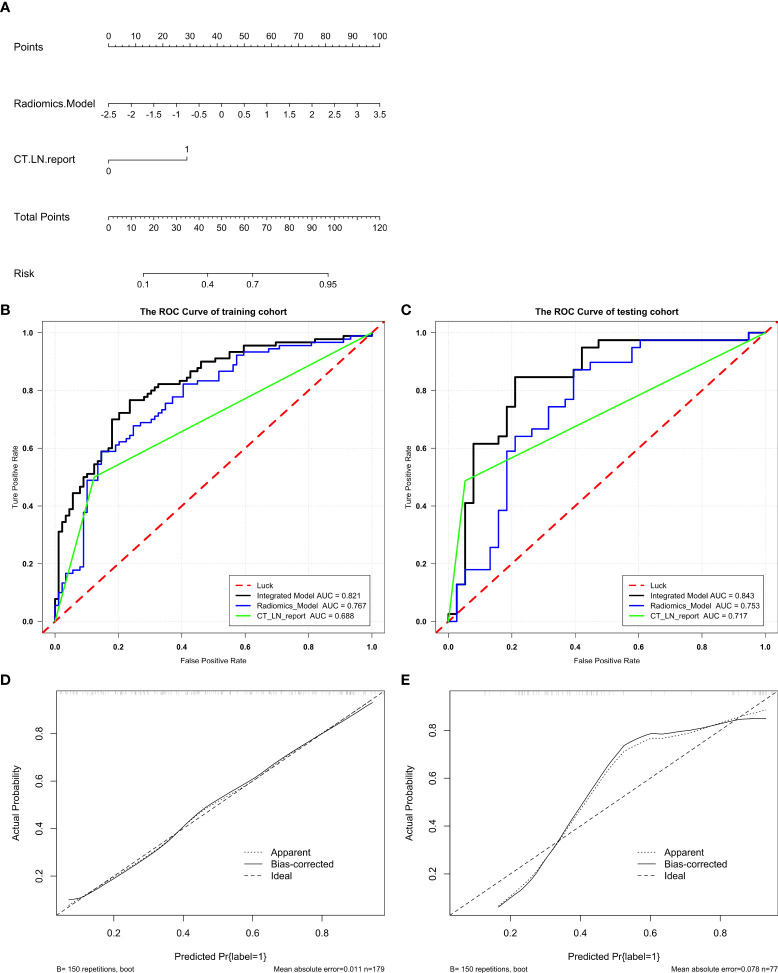
Radiomics nomogram for the diagnosis of LN metastasis in patients with HGSOC. A radiomics nomogram was developed in the training **(A)**. Comparison of ROC curves among the Integrated Model, Radiomics Model, and CT_LN_report alone for the prediction of LN metastasis in the training **(B)** and test cohorts **(C)**. Calibration curves of the radiomics nomogram in the training **(D)** and test cohorts **(E)**. Good alignment of the diagonal dashed reference line and solid line indicates good performance.

An Integrated Model that incorporated the Radscore and CT_LN_report was developed in the training and test cohorts and presented as a nomogram **Figure 4A**. The Integrated Model yielded an AUC of 0.821 (95% CI: 0.760, 0.882) in the training cohort and 0.843 (95% CI: 0.750, 0.936) in the test cohort, showing favorable predictive efficacy (**Table 2**).

All ROC curves are shown in **Figures 4B, C**. There were no differences between the Radiomics Model and CT_LN_report in the training (P=0.093) and test cohorts (P=0.619), and a significant difference was present between the Integrated Model and CT_LN_report and between the Integrated Model and Radiomics Model in the training cohort (P=0.004) and the test cohort (P=0.019).

The calibration curve of the Integrated Model demonstrated good agreement between the predicted and observed LN metastasis rates in the training cohort (**Figure 4D**) and the test cohort (**Figure 4E**).

### Validation of the Combine Model in the CT-Reported LN-Negative Subgroup

**Figure 5:**
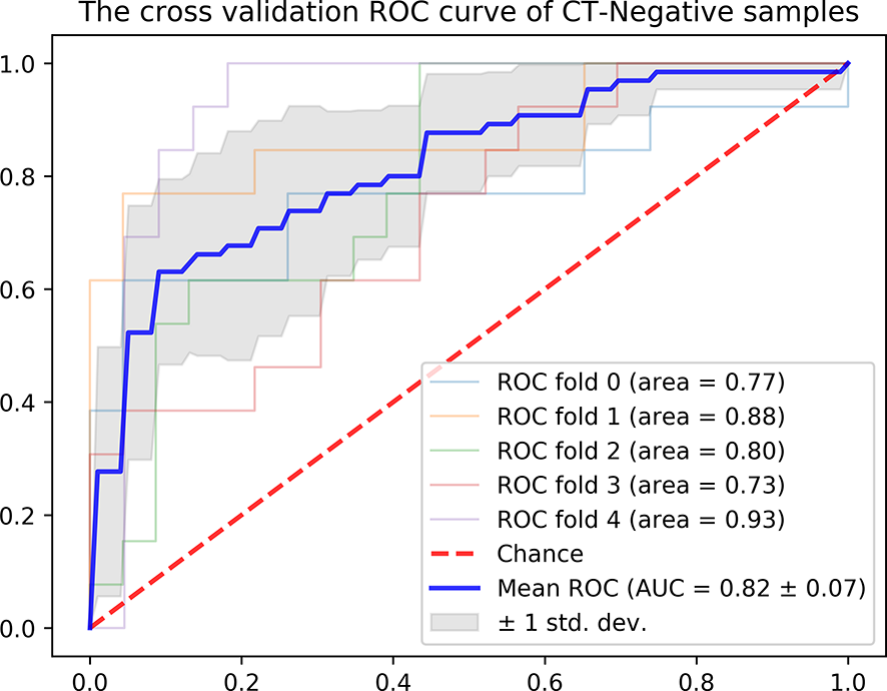
ROC curve of the radiomics nomogram in the CT-reported LN-negative samples by five-fold cross-validation.

In addition, we evaluated the discriminative efficiency of the Integrated Model nomogram in the CT-reported LN-negative subgroup (n =179) using ROC analysis by 5-fold cross-validation. The Integrated Model nomogram yielded an average AUC of 0.82 (**Figure 5**).

### Clinical Use of the Radiomics Nomogram

**Figure 6:**
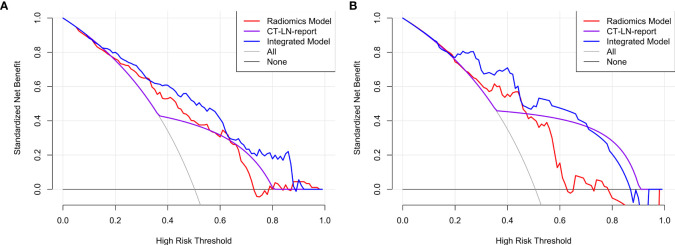
The DCA for the Integrated Model, Radiomics Model, and CT_LN_report in training **(A)** and test cohorts **(B)**.

The DCA for radiomics nomogram, Radiomics Model, and CT_LN_report in the training and test cohorts are presented in **Figure 6**. The DCA showed that the radiomics nomogram adds more net benefit to predict LN metastasis than either the Radiomics Model or the CT_LN_report alone for more than 5–10% when the threshold probability is within a range from 0.34 to 0.6 in the training and from 0.2 to 0.46 in the test cohorts.

## Discussion

In our study, we constructed and validated an Integrated Model that included the Radscore and CT_LN_report for the prediction of LN metastasis in patients with HGSOC before surgery. Compared with the Radiomics Model and CT_LN_report, the Integrated Model showed excellent discrimination in both the training cohort (AUC, 0.821) and the test cohort (AUC, 0.843). Therefore, a radiomics signature could be used to assist clinical and traditional CT images to improve the diagnostic value for LN metastasis in HGSOC.

Contrast-enhanced CT is the standard imaging method for the preoperative evaluation and postoperative surveillance of women with ovarian cancer, and LN metastasis has been evaluated to measure LN size (22). Unfortunately, our results demonstrated that the AUC values of the CT_LN_report were 0.688 and 0.717 with sensitivities of 50 and 48.7% in the training and testing cohorts, respectively. Therefore, 50.4% of patients with LN metastasis were understaged, and 10.2% of patients without LN metastasis were overstaged according to the CT visual images, which was similar to the findings of several previous studies (9, 24, 25). In view of CT_LN_report results, the discrimination of malignancies from benign nodes on the basis of morphological features remains challenging because of small LN metastasis and non-specific inflammatory hyperplasia (35).

Radiomics is the process of the conversion of medical images into high-dimensional, mineable data *via* the high-throughput extraction of quantitative features, followed by subsequent data analysis for decision support (27) and is mainly used in oncology to facilitate improved clinical decision-making (28). Therefore, we first extracted 696 radiomics features from each ROI, and nine LN status-related features with non-zero coefficients in the random forest model were selected based on the training cohort. However, the Radiomics Model achieved AUC values of 0.767 and 0.753 with sensitivities of 58.9 and 60.5% in the training and test cohorts, respectively, which were similar to those of the CT_LN_report (P>0.05). Thus, compared with contrast-enhanced CT, the Radiomics Model alone does not provide more diagnostic value.

Therefore, to provide a convenient and non-invasive tool for clinicians, we constructed a radiomics nomogram based on Radscore and CT_LN_report, which showed good calibration and discrimination in the training cohort (AUC, 0.821) and the test cohort (AUC, 0.843). Our results demonstrated that the radiomics nomogram had better predictive efficacy than either the Radiomics Model or the CT_LN_report alone. Furthermore, our radiomics nomogram is good for discriminatory ability in the CT-LN-reported negative subgroup, with an average AUC of 0.82 by five-fold cross-validation. Additionally, the radiomics nomogram adds more net benefit to predict LN metastasis than either the Radiomics Model or the CT_LN_report alone for more than 5–10% when the threshold probability is within a range from 0.34 to 0.6 in the training and from 0.2 to 0.46 in the test cohorts. Therefore, compared with contrast-enhanced CT and Radiomics Model, the predictive power of the radiomics nomogram is clearly superior and may serve as an accurate and reliable predictive tool for LN metastasis in patients with HGSOC. At present, for a general gynecologist, it is difficult to understand the mechanism of radiomics, but the calculation design part can be completed with the help of the computers. Furthermore, radiomics can be further developed into the application-oriented software to assist clinicians in working, especially for tumor staging evaluation and the selection of an appropriate treatment strategy preoperatively.

In addition, nine LN status-related features were selected from 696 radiomics features by RF. Local binary patterns (LBP), and LBP is an effective texture descriptor for images that thresholds the neighboring pixels based on the value of the current pixel (36, 37). LBP descriptors efficiently capture the local spatial patterns and the gray-scale contrast in an image. Original_glcm_ClusterProminence, and glcm describes the second-order joint probability function of an image region constrained by the mask. Cluster Prominence is a measure of the skewness and asymmetry of the glcm, and a higher value implies more asymmetry about the mean, while a lower value indicates a peak near the mean value and less variation about the mean. Original_glszm_GrayLevelNonUniformityNormalized, and glszm quantifies gray-level zones in an image, and Gray Level NonUniformity Normalized (GLNN) measures the variability of gray-level intensity values in the image, with a lower value indicating a greater similarity in intensity values. Original_shape_Maximum2DDiameterColumn, and Shape_Maximum 2D Diameter Column is defined as the largest pairwise Euclidean distance between tumor surface mesh vertices in the row-slice (usually the coronal) plane (38).

Our study has several limitations. First, it included a relatively small number of patients recruited in a single center. Although the results indicate that the radiomics nomogram has substantial value in the preoperative evaluation of LN status in patients with HGSOC, the reliability of the radiomics nomogram for diagnosing LN metastasis in HGSOC needs to be further investigated in multiple centers. Second, all images were manually segmented, which may have resulted in inconsistent, subjective tumor segmentation and could reduce the model’s performance. Furthermore, we were unable to obtain genomic features in all patients with HGSOC due to the retrospective nature of the study, so we could not analyze the relationship between LN metastasis and genomic features.

In conclusion, we constructed and validated a radiomics nomogram that incorporated the Radscore and CT_LN_report to predict LN metastasis in patients with HGSOC preoperatively. The radiomics nomogram had a better discrimination ability in both the training cohort and the test cohort than the Radiomics Model and CT_LN_report alone, which greatly improves the diagnostic efficiency and could be recommended as a new, convenient, and non-invasive method to predict LN metastasis.

## Data Availability Statement

The original contributions presented in the study are included in the article/**Supplementary Material**. Further inquiries can be directed to the corresponding authors.

## Ethics Statement

The studies involving human participants were reviewed and approved by the Institutional Review Board of West China Second University Hospital (No. 2020173). Written informed consent for participation was not required for this study in accordance with the national legislation and the institutional requirements.

## Author Contributions

Guarantor of integrity of the entire study: Y-KG and GN. Study concepts and design: H-ZC, X-RW, and Y-KG. Literature research: H-ZC, GN, and F-MZ. Clinical studies: H-ZC, GN, F-MZ, X-SL, and X-JC. Experimental studies/data analysis and statistical analysis: H-ZC and X-RW. Manuscript preparation: H-ZC and X-RW. Manuscript editing: H-ZC. All authors contributed to the article and approved the submitted version.

## Funding

This study was supported by Sichuan Science and Technology Program (No.2020YFS0050), West China Second University Hospital, Sichuan University (KL007).

## Conflict of Interest

The authors declare that the research was conducted in the absence of any commercial or financial relationships that could be construed as a potential conflict of interest.

## Publisher’s Note

All claims expressed in this article are solely those of the authors and do not necessarily represent those of their affiliated organizations, or those of the publisher, the editors and the reviewers. Any product that may be evaluated in this article, or claim that may be made by its manufacturer, is not guaranteed or endorsed by the publisher.
