# Supplementary material for: The Development and Validation of a CT-Based Radiomics Nomogram to Preoperatively Predict Lymph Node Metastasis in High-Grade Serous Ovarian Cancer
Source: Front Oncol. 2021 Aug 31;11:711648. doi: 10.3389/fonc.2021.711648 (PMC8438232; doi:10.3389/fonc.2021.711648)
Supplement: Supplementary file 1 [file Table_1.docx]

Appendix E1 The distributions of the Radscore for each patient in the training cohorts

| label | Radscore | PLBP_hist_tumor_orient4_5 | original_shape_Maximum2DDiameterColumn | original_glcm_ClusterProminence | WL_lbp_hist_cV1_1 | WL_lbp_hist_cV2_1 | WL_lbp_hist_cV2_8 | WL_lbp_hist_cH2_7 | WL_lbp_hist_cH2_0 | original_glszm_GrayLevelNonUniformityNormalized |
| --- | --- | --- | --- | --- | --- | --- | --- | --- | --- | --- |
| 1 | -0.113268377 | -0.345338434 | 0.128956772 | 0.806703956 | -0.052601769 | -0.184233832 | 1.078705402 | -0.176190363 | 0.259836649 | -1.138485295 |
| 1 | 0.604081771 | -0.471784867 | 0.809983531 | -0.353352113 | -0.370327538 | 0.276440524 | 0.280445081 | 0.267397442 | 0.332544979 | 0.002142817 |
| 1 | -0.975186739 | -0.553738964 | 0.014394902 | -0.786901833 | 0.558049032 | -1.254724952 | 1.2688964 | -0.183914483 | 0.893571138 | 1.079196093 |
| 0 | 0.541761966 | -0.944022284 | -1.602333847 | 0.133300905 | -0.037066847 | 1.285930391 | -1.352570234 | -0.773602178 | -1.202831156 | -0.299796869 |
| 0 | 1.725856145 | -0.651241775 | 0.002307421 | -0.06937659 | -0.733106727 | 0.370509784 | -0.844026642 | 1.772343683 | -1.362698045 | -0.234738244 |
| 0 | -1.755781383 | 5.981355588 | -2.069110325 | -0.667881044 | -0.134940986 | 5.892480811 | -5.096140955 | 0.958588418 | 3.395843848 | 0.538928904 |
| 0 | -0.655248695 | -0.211050668 | -1.123251397 | 0.368028983 | -0.982877739 | 0.192669982 | 0.227068555 | -0.252844365 | -1.161213745 | -0.395621213 |
| 0 | -1.743474086 | -0.201862444 | -1.155368866 | 1.05971413 | -0.547549949 | -0.521636586 | 0.690661876 | -0.99723656 | 0.25435421 | -1.102654646 |
| 1 | 0.693504334 | 0.761286108 | -0.275305141 | -0.333165281 | 1.277981255 | 4.287877597 | -0.651594703 | -3.947294042 | 0.036147793 | -0.392057501 |
| 1 | -0.275333511 | -0.261843552 | 0.175136275 | 0.241326969 | -0.678921334 | -0.351871219 | 0.71179096 | 0.500983741 | -0.474390217 | 0.616524993 |
| 1 | 0.490641285 | -0.997147175 | -0.625911543 | 0.822539423 | -0.505205704 | -0.529032515 | 0.646019023 | 0.749065557 | -1.378996426 | -0.92733402 |
| 1 | 0.650205422 | -0.148321833 | 0.547714264 | -0.257451994 | 0.398840497 | -0.601088597 | -0.192022882 | -0.035624574 | -0.049816834 | -0.335173562 |
| 1 | 0.219032369 | -0.785969353 | -1.331664386 | -0.867573376 | -0.390065026 | 0.799967779 | -0.515939016 | 1.564214207 | -1.653386214 | 3.281327702 |
| 0 | 0.220922557 | 3.86888358 | -0.031839247 | -0.134160117 | 1.040097179 | 1.340828582 | -2.548124839 | -0.935557344 | -1.112836706 | 0.204356524 |
| 0 | -2.024364409 | -0.879071635 | -0.005353116 | -0.664323865 | -0.774475337 | -0.484548576 | 1.113707699 | -0.848099993 | 0.680801015 | 2.88773983 |
| 0 | 0.046642596 | -0.151185705 | 0.191351958 | -0.725620745 | 0.183439583 | -0.358558754 | 0.138737515 | -0.521656538 | -0.429249854 | 0.695881378 |
| 1 | 1.435844819 | -1.10969352 | -0.212977999 | 0.412637468 | -0.756891484 | 1.48784441 | 0.660154051 | -0.190753961 | -1.953732528 | -0.821152517 |
| 1 | 2.503924738 | -1.134882222 | -1.534789223 | 2.144294857 | -0.298425091 | 1.060041083 | -0.596221207 | 2.329126912 | -3.187219273 | -1.603235269 |
| 1 | -1.801621859 | 1.728584088 | -1.576797286 | -0.755125695 | 0.811769975 | -0.353231203 | -0.505533041 | -0.985447278 | 0.058813563 | 0.485628114 |
| 0 | 0.405441425 | -0.838825576 | 0.009228955 | -0.240972076 | -0.63579293 | -0.048731288 | -0.418706668 | 0.822580966 | -0.322006395 | 0.740333757 |
| 1 | 0.782919796 | -0.896683257 | 0.160633796 | -0.476717447 | 0.972929462 | 0.519966802 | 0.021286537 | -0.141838753 | 0.954228455 | 0.110925934 |
| 1 | 0.291978334 | -0.547142152 | 0.338336913 | 0.464340386 | -0.267510222 | -0.133804135 | 0.633351684 | -0.122883492 | 0.043974879 | -1.036367067 |
| 0 | -0.963221209 | 1.249079726 | -0.723530614 | 0.313722427 | 0.547291746 | -0.311861192 | 1.149948883 | -1.165521216 | -0.935493742 | -0.712095265 |
| 0 | 0.1408136 | -0.716093749 | -1.39466295 | -0.723948932 | 1.383525036 | -0.405203882 | 0.086048093 | 0.419515059 | -0.406520726 | 1.028912547 |
| 0 | -1.161285428 | 1.278982548 | -0.084641705 | -0.143051251 | 0.01029326 | -0.746916077 | 0.405554218 | 0.299342127 | 0.822502083 | -0.309197132 |
| 1 | 0.767075275 | -0.075618784 | -0.132353356 | -0.544080953 | 0.331204974 | -0.088279015 | -0.096852536 | 1.015601986 | -0.226438313 | -0.044908234 |
| 0 | -0.018229961 | -0.709140777 | 0.994124272 | 0.148109642 | -0.342441398 | -0.541674896 | 0.946555517 | 0.179057732 | 1.272753897 | -0.704221225 |
| 1 | 0.516905149 | 1.032348762 | 1.949531312 | -0.601828259 | -0.522837586 | -0.015565751 | 0.153156565 | 0.245086891 | 0.50908967 | -0.041103034 |
| 0 | -0.633080726 | 1.301113724 | 2.901315622 | -0.624984511 | 0.436986019 | -0.687677858 | 0.773042056 | -0.523749624 | 1.47553089 | 3.013462567 |
| 1 | 0.830401538 | -0.481435509 | -1.148765261 | -0.321784425 | 1.982486142 | 0.493460834 | 0.103064572 | 0.058774583 | 0.209307726 | -0.375068215 |
| 0 | -1.338954825 | 2.012478829 | 0.683482866 | -0.603753923 | -0.005457153 | -0.58251549 | 0.492973813 | -0.575677433 | 0.924705273 | 0.000518333 |
| 0 | 0.369807053 | -0.704730025 | 0.508695571 | -0.467983814 | -0.939984038 | -0.685675723 | 0.846638897 | -0.002801059 | -1.119126086 | -0.248549264 |
| 1 | 1.153969066 | 0.040935936 | 0.360763647 | 0.389745696 | -0.155553567 | 0.137089266 | -1.283376363 | 0.05713046 | -0.781909274 | -0.854248591 |
| 0 | 1.011759197 | -0.95235531 | 1.004648355 | 0.400332706 | 0.142430552 | -0.405841664 | 1.14775404 | 0.583406599 | 0.450651991 | -1.071885869 |
| 0 | -0.347671629 | -0.495207406 | 0.237554009 | 0.361199209 | -0.681723044 | -0.951423871 | 0.425216108 | 0.391215217 | 0.464753196 | -0.857610009 |
| 0 | -0.332191173 | -0.923030427 | -0.341653594 | 0.054426694 | -0.959520122 | -0.100459271 | 1.001748786 | 0.03632102 | -0.433902035 | -0.31517796 |
| 0 | 0.289998711 | -0.589517205 | 0.137996446 | -0.042127739 | -0.020792273 | 0.014083334 | 0.751577798 | 0.822530113 | 0.941790818 | -0.793731836 |
| 1 | 0.30802492 | 0.072564366 | 0.668607863 | 0.059628646 | -0.204171402 | -0.123915106 | 0.637308648 | 0.74560939 | 0.371201368 | -0.31344301 |
| 1 | -0.805837082 | -0.670417651 | 0.003050255 | -0.752396065 | -0.797626748 | -0.577994911 | 0.312629671 | -0.446245229 | 0.12135712 | 0.784845202 |
| 0 | 0.475371246 | -0.302469047 | 0.521348602 | 0.541196914 | -0.54411041 | 0.018330378 | 0.851675041 | 1.552451492 | 0.72214558 | -1.038777818 |
| 0 | -0.122860315 | -0.272423733 | -0.352098487 | 0.58514482 | 0.49965403 | -0.7149284 | -0.757090428 | -0.234947063 | 0.322917475 | -0.495447826 |
| 0 | 0.30455965 | 2.596866644 | 0.133999132 | 0.066647093 | 0.145565906 | 0.170998678 | -1.601493214 | 0.538265548 | -1.321952155 | 0.270402706 |
| 0 | -1.884007449 | 2.294522362 | -1.298419615 | 0.400087457 | -0.200736025 | -1.773890505 | -0.662562872 | -0.278297866 | -1.081549118 | -0.865592919 |
| 0 | 1.278511249 | -0.16884772 | -1.660667158 | -0.209832287 | 2.363928882 | 1.234633598 | -2.288820468 | -1.029140355 | -0.619158394 | 0.091303482 |
| 0 | -0.199007533 | -1.085576659 | 0.078927987 | 0.801568634 | 0.2148261 | -1.504474193 | 0.227741184 | -0.571213745 | 0.248585374 | -0.93991426 |
| 0 | -0.203759905 | -1.09922886 | -1.17097913 | 0.954773409 | -0.938402273 | 0.176371672 | 0.580370607 | -0.057088259 | -1.18620121 | -0.980243022 |
| 0 | -1.078433393 | -0.603901356 | -0.605538855 | -0.41131707 | 0.41804678 | -1.733578803 | -0.380989665 | -0.55363298 | -0.014112678 | 1.361017939 |
| 1 | 0.510720629 | 1.198115314 | 3.268967278 | -0.64376971 | 0.261163503 | -0.53085812 | 0.837939625 | -0.235840008 | 1.157472278 | 1.110559293 |
| 1 | 0.572856768 | 0.681804688 | -1.267547792 | 0.178242074 | 0.146070854 | 1.251140424 | -0.504054149 | 0.205370346 | -1.522117515 | -0.610021433 |
| 0 | 0.283799654 | 0.789511075 | 0.34737847 | -0.304103445 | 0.071526475 | -0.365249086 | 1.066147707 | 0.313549128 | -1.428827424 | 0.400783254 |
| 0 | -0.400818399 | -1.013172369 | 0.75420916 | -0.679638407 | -1.274442901 | -0.746148432 | 1.576094055 | 0.14327247 | -0.975333563 | 1.875074031 |
| 1 | -0.142126978 | -0.692195594 | -0.299838988 | 0.770920672 | 0.026826551 | -0.089503368 | 0.590327304 | 0.104622394 | 0.756234145 | -1.229836303 |
| 1 | 0.855369712 | -0.567420825 | 0.380274205 | 0.835573068 | 0.400583961 | -0.025259198 | -0.775230401 | -0.499722925 | 0.206250872 | -1.178363408 |
| 0 | -1.013804757 | 2.213141933 | 0.193398205 | -0.852026991 | 0.454589724 | 0.147824243 | 0.201795441 | -0.51607615 | 0.123437099 | 1.067049322 |
| 0 | 0.287339941 | -0.946000434 | 1.135497823 | 0.425657964 | -0.910146947 | -0.571914571 | 1.349654239 | -0.058779085 | -0.503330473 | -0.11558574 |
| 1 | 2.24605426 | -0.671614082 | -1.791755623 | -0.796166407 | 1.067652237 | 1.726586187 | -1.533791917 | 3.448963495 | -0.686191401 | 1.21271302 |
| 0 | -2.212246716 | -1.040967266 | 0.353672841 | 11.00143026 | -1.227477595 | -0.13750778 | 1.514779811 | -0.279395055 | -0.336076593 | -0.859537602 |
| 0 | -1.011737325 | 0.165567781 | 0.447379124 | -0.168766924 | -1.144586361 | -0.88405179 | 0.666094998 | 0.285440662 | -0.059950355 | 0.79697801 |
| 0 | -1.123973244 | -0.379855068 | 0.750866827 | -0.737497748 | 0.075552292 | -0.367294742 | 1.104222586 | -0.126306656 | 0.570890499 | 3.73100056 |
| 1 | 0.433805337 | -0.695996636 | 0.735632586 | 0.550339404 | 0.209387581 | -0.302960035 | -0.098644397 | -0.248356807 | 0.832115182 | -0.663892623 |
| 1 | -2.467098716 | 2.157287552 | -1.493779787 | -0.764897413 | 0.566245715 | -1.866792393 | -0.704797445 | -1.046967915 | -0.244807273 | 0.400507567 |
| 1 | 0.808525103 | -1.025376509 | -0.068224455 | -0.564648198 | 0.374007419 | 0.418008376 | 0.024250864 | 0.497743442 | 0.154867275 | 0.619560648 |
| 1 | 1.155341234 | 0.845353025 | 0.465340445 | 0.181313872 | 0.276146728 | 0.133698307 | -0.932115793 | 1.184787059 | -0.293267203 | -0.501158397 |
| 0 | -1.951370674 | 1.198169402 | 0.082081142 | -0.869054938 | -0.942137032 | -0.216526854 | 1.359401861 | 0.868637173 | 0.687090273 | 2.134025005 |
| 1 | -0.453336015 | 0.38474013 | 0.002521473 | -0.531795386 | 0.200318365 | -0.018879106 | 0.028533683 | -0.200318354 | 0.308502649 | 0.805958652 |
| 0 | -0.81284713 | 0.007938586 | -0.306159193 | -0.344741106 | -2.484507245 | 1.101763631 | -2.394569681 | -0.164644969 | 0.794340691 | -0.355737373 |
| 1 | 1.383114034 | -1.108748863 | -0.377390577 | 0.918261671 | -0.016590029 | -0.115931558 | 0.085142695 | 0.504456513 | -1.554312027 | -1.292630256 |
| 1 | 0.044863128 | -0.095955864 | -1.188971565 | 0.422740042 | 0.909828227 | -0.674862268 | 0.549405275 | 1.621335916 | 0.301497521 | -0.861479226 |
| 0 | 0.56693205 | -1.054140107 | 0.372156072 | 0.176985168 | -0.606567091 | -0.620615143 | 1.385788358 | 0.023145825 | -1.303481503 | -0.509202358 |
| 1 | 0.654284321 | 0.072121625 | 1.21298591 | 0.408938899 | 2.50711772 | -0.526106304 | 0.722389158 | -0.215354163 | 2.233315678 | -0.359338455 |
| 1 | 0.472493181 | 0.210914307 | -0.003705232 | -0.226376869 | 0.121167014 | -0.165549039 | -0.900901732 | -0.185942187 | -0.558743648 | -0.225249804 |
| 0 | -1.205842554 | -0.80042607 | -0.281205221 | 0.049098208 | -0.581344016 | 1.38853901 | -0.569037172 | -2.649408317 | 1.78054611 | -0.610573262 |
| 1 | 1.191032351 | -0.617332403 | 0.507792448 | -0.196068922 | 0.562303149 | -0.68141924 | 0.895607198 | 0.434870518 | -1.106993402 | 0.019955558 |
| 0 | 0.075370143 | 1.248556117 | 0.024583119 | -0.5902981 | -0.112127061 | 0.231553535 | -0.647987388 | -0.181057557 | -0.650335867 | -0.089954785 |
| 0 | -0.058259477 | 0.129319298 | 0.024438276 | 0.175830193 | 1.558053642 | -0.484588601 | 0.548527635 | -0.085356551 | 1.581414269 | -0.86903348 |
| 0 | -0.907734725 | -0.429942399 | -0.923570464 | -0.217145354 | -0.755975405 | -0.172494029 | 0.235610401 | -0.008516364 | 0.012004504 | -0.125291348 |
| 0 | -1.034954149 | -0.829741211 | -1.099362408 | 0.20291668 | -0.470534301 | -0.65994544 | 0.515818226 | -0.089144822 | 0.498133215 | -0.975247082 |
| 0 | 1.13738021 | 0.601935624 | 0.409914787 | -0.397396339 | -0.14009133 | 2.073675747 | -0.892334011 | -0.172715516 | -0.127158423 | -0.180119844 |
| 1 | 0.284079678 | 0.033613078 | 0.465617272 | 0.148196667 | -0.345085572 | -0.141705885 | -0.009876867 | 0.187646984 | 0.04911159 | -0.602491134 |
| 0 | 1.134952551 | -0.556699111 | -0.035623744 | 0.085343291 | -0.526694019 | -0.819502974 | -0.966979202 | 0.643363441 | -1.566402106 | -0.790311443 |
| 0 | -1.122999303 | 2.434693227 | -1.301307656 | -0.074985784 | 0.087580754 | 1.811600657 | -1.454470143 | -1.33929078 | -0.557621866 | 0.299640172 |
| 0 | -1.60170809 | 1.694487109 | -0.691327916 | -0.770499755 | -0.392884026 | 1.296016051 | 0.284654639 | -0.877696494 | -0.023826126 | 1.339907181 |
| 0 | -0.333183396 | -0.355051419 | -0.230065675 | 0.327335073 | -0.346815587 | -0.096238269 | 0.423713622 | 0.490436528 | 0.506012917 | -0.360795893 |
| 0 | 0.970300129 | -0.769971611 | 0.112672839 | -0.500208346 | 8.561393532 | -3.043311404 | -1.963225586 | -5.002897112 | 3.227100728 | 0.132776853 |
| 1 | 0.150723995 | 0.242005449 | 0.672885651 | -0.50098164 | -0.668783306 | 0.105308182 | -0.352332784 | -0.183718127 | -0.011582228 | -0.007666139 |
| 1 | -0.095742597 | -0.46212573 | -0.031234407 | -0.583470861 | -0.596155937 | -0.498769069 | -1.173683967 | -0.954541663 | -0.723060311 | 0.447820411 |
| 1 | 1.483833119 | 0.702078225 | 0.877656354 | 0.975270744 | 0.914468499 | 0.261737455 | -0.022434165 | 0.728673738 | -0.124509312 | -1.203364099 |
| 0 | -0.53490212 | -0.963396613 | -0.550554888 | 0.246410805 | -0.015967248 | 0.288721104 | 0.178432851 | -0.37969944 | 1.095210235 | -0.275664806 |
| 0 | 0.375487623 | -0.388737621 | 0.414224993 | -0.576430513 | 0.171685529 | -0.182018112 | 0.10826062 | 0.064286682 | 0.388581001 | 0.074521508 |
| 1 | -0.337128624 | -0.658720564 | -0.838392337 | 0.51812075 | -0.471679961 | -0.235816045 | 0.32572428 | -0.148141883 | -0.604357677 | -0.712258227 |
| 0 | -1.812610236 | 0.898723979 | -0.153651182 | -0.761386276 | 0.503250145 | -0.673703634 | 0.997805998 | -0.599630031 | 1.391745492 | 0.909018901 |
| 1 | -0.432945815 | -0.620164887 | 1.86762996 | -0.255823316 | -0.165454971 | -0.80804999 | 0.701630739 | -0.287278793 | 1.840669087 | 1.220546246 |
| 1 | 0.271397648 | 0.441234265 | 1.047263368 | -0.181732467 | -0.730635779 | -0.665069544 | 0.733773396 | -0.376152568 | -1.419997705 | -0.052137249 |
| 0 | -0.764723875 | -0.665100108 | -0.60145145 | -0.276337914 | -0.505746514 | -1.488657234 | -1.346862154 | -1.063425752 | -0.15333872 | -0.510009957 |
| 1 | -0.214518928 | -0.120780919 | 0.870180243 | -0.776691058 | 0.702848453 | -0.351061976 | 0.861455477 | 0.139842457 | 0.928658186 | 1.954193195 |
| 0 | -0.328764344 | -0.606939828 | 0.138197596 | 0.06540488 | -0.254094353 | -0.373169712 | 0.853351707 | 0.075909913 | 0.90773143 | -0.754260705 |
| 0 | 0.363591591 | 0.076371311 | -1.107495816 | -0.017826339 | -0.269367035 | 0.406010419 | 0.11655116 | 1.063558243 | -0.923272428 | -0.815657449 |
| 1 | 1.771543753 | 1.420191129 | 3.886141437 | -0.548759517 | 0.347493956 | -0.323695378 | -0.098130482 | -0.136620774 | 0.825992562 | -0.045832505 |
| 1 | -0.527388874 | 0.938797597 | 0.176917411 | -0.256176098 | 0.005610402 | -0.081826738 | 0.413842775 | -0.000569526 | 0.446382771 | -0.182099362 |
| 0 | -0.124649598 | 0.71380406 | -0.000371452 | -0.144703988 | 0.138615744 | -0.390552199 | 0.326222234 | 0.293744926 | 0.014786071 | -0.609049937 |
| 0 | -0.664871983 | 0.308136596 | 0.161809382 | 0.344127357 | -1.325117031 | -0.759439256 | -0.641616593 | -0.640884841 | -0.691855371 | -0.63248489 |
| 1 | -1.702957832 | 0.598896768 | -1.367462526 | 0.48399333 | -0.256203161 | 0.296720033 | 0.010450614 | -2.356816655 | -0.700894943 | -0.564139312 |
| 0 | -1.212441014 | -0.834523433 | 0.500741696 | -0.655828912 | 0.08344511 | -0.806867934 | 0.536029817 | -0.518567762 | 1.430285295 | 2.189430563 |
| 0 | -1.244059821 | -0.299251175 | -0.759407978 | -0.851620831 | 0.265962913 | -0.473155192 | -0.06565731 | -1.092830878 | 0.279832798 | 1.328852707 |
| 1 | 1.569210734 | 0.831446842 | 0.735553836 | 0.763573656 | 0.77805948 | 0.481819084 | -2.15250157 | -0.316362401 | -1.026606556 | 0.000532113 |
| 0 | -0.496270374 | 1.501199177 | 0.625112438 | -0.465342312 | 0.272701105 | -0.258624136 | 0.503546435 | -0.192053734 | 0.531857863 | -0.166724886 |
| 0 | -0.687938951 | 1.25514021 | -1.956245934 | -0.281246683 | -0.891428641 | -1.473470367 | -2.062763908 | 2.451188894 | -1.090790609 | -0.472805528 |
| 1 | 1.592115855 | -0.525638012 | -0.269519082 | -0.027764585 | -0.519178392 | 0.684397627 | -0.456559287 | 0.583283928 | -2.345295302 | -0.092102398 |
| 1 | 0.911799906 | -1.020406631 | -0.093851066 | 1.307965554 | -0.824877941 | 0.621308945 | 0.224033049 | 0.04259177 | -1.066174386 | -1.511157903 |
| 0 | 1.486729574 | 0.809344847 | -1.46375416 | -0.74293053 | -0.010685625 | 2.767058732 | -2.966109338 | 1.956103148 | -0.481211129 | 0.633722732 |
| 0 | -0.478617243 | -0.975758459 | -0.204190133 | 0.759469259 | -1.267850077 | -0.200659558 | 1.013156022 | -0.23555087 | -0.629699414 | -0.620377359 |
| 1 | 0.95873626 | -1.103333269 | -0.2539668 | 0.710679399 | -0.530736807 | 0.302958901 | 0.076916494 | 0.087241307 | -1.286114689 | -0.906882865 |
| 0 | -1.629562533 | 0.886821096 | -0.242644839 | -0.516906972 | -0.408653011 | 0.114689991 | 0.434420471 | 0.133943872 | -0.101614889 | 3.033273647 |
| 1 | 0.824915122 | -0.301772133 | 0.24159731 | -0.401658331 | 0.438514312 | -0.345830031 | -0.314404327 | 0.720916407 | 0.357047791 | -0.433201847 |
| 0 | -0.368818447 | -0.837741978 | 0.147056535 | -0.467965778 | 0.015630278 | 0.108178812 | 0.749021013 | 0.220055785 | 0.805297145 | 1.460164416 |
| 1 | 0.313489164 | -0.50228804 | 0.008013225 | 0.025599629 | -0.379950692 | 0.17654698 | 0.603756791 | 0.432424156 | -0.03787911 | -0.578992483 |
| 1 | -0.464227708 | -0.456357816 | -0.375200214 | 0.525979097 | -0.497013503 | -0.261863558 | 0.450786329 | -1.042495302 | -0.507925398 | -1.047476363 |
| 0 | -0.360280392 | 1.758851181 | -0.573526542 | -0.515168535 | 2.56947386 | -2.478864727 | 0.114387445 | 1.449605265 | 0.517825876 | 0.176484349 |
| 1 | -0.060552824 | -0.450045559 | 0.502595823 | -0.815470427 | -0.826533352 | -0.758184142 | -1.265076684 | -0.143425588 | -0.406023988 | 1.427410418 |
| 1 | 0.539435337 | -0.469112393 | 0.409507801 | -0.451631792 | -0.062070909 | -0.356621638 | 0.424855357 | 0.625154169 | -0.248790828 | 0.383853399 |
| 0 | -0.03312566 | -0.041962194 | -0.650926067 | -0.603897062 | -0.445548171 | 0.873868517 | 0.375637633 | 0.638715436 | -0.232887006 | 0.33629544 |
| 0 | -0.144052573 | -0.147898578 | 1.627731718 | 0.931483837 | -0.162786142 | -0.433023866 | 0.7806561 | -0.296306215 | 1.122344578 | 0.000983537 |
| 1 | 1.114949422 | -0.487848073 | 1.74294339 | -0.246332486 | 0.753367608 | -0.549089527 | 0.851248762 | 0.219823935 | 1.061331157 | -0.364589412 |
| 1 | -0.054462112 | 1.325690076 | 0.188729679 | 0.622188384 | 0.191400922 | 0.572859362 | 0.068152983 | -0.12357842 | 0.28305043 | -1.140510441 |
| 1 | 0.686289114 | 0.150879351 | -1.24442089 | 0.399855512 | -0.490409793 | 1.622954566 | -0.091700305 | 0.469176879 | -1.787300483 | -0.570993581 |
| 0 | -0.412806418 | -0.604475167 | -1.582539927 | -0.919434442 | -0.327485709 | 3.124567087 | -2.079262903 | -3.169578928 | -0.864325314 | 1.702602874 |
| 1 | 0.484720168 | -0.068675699 | 0.506945328 | -0.118803648 | -0.522820615 | 0.015274828 | -0.482787961 | 0.000564817 | -0.568491766 | 0.130123199 |
| 1 | -0.023081885 | 1.217371387 | 1.121580024 | -0.323155512 | 0.492878054 | -0.052258403 | 0.226195376 | -0.152466709 | 1.040115075 | -0.166710012 |
| 0 | -2.151402544 | 0.607046363 | -1.505564315 | -0.79171988 | -0.272285297 | -0.128193437 | 0.046540518 | -0.06558639 | 0.803841721 | 1.181373388 |
| 0 | -1.408101848 | 0.073049725 | 0.226516775 | 1.018978616 | 0.148410202 | -0.608755614 | 1.075240841 | -0.476881008 | 2.104752124 | -1.003756798 |
| 1 | 1.002730223 | -0.398575006 | -0.011975903 | 0.789264584 | 0.708664483 | -0.333797991 | -0.24552174 | 0.718590017 | -0.199789599 | -0.804834699 |
| 1 | 0.74866708 | -0.517238515 | 1.282338548 | 0.359291041 | 0.291121308 | -0.736998384 | 0.722851324 | 0.517768078 | 1.079908018 | -1.09853858 |
| 1 | -0.607992471 | -0.052976477 | -0.488161149 | 0.090836145 | -0.201778902 | -0.687334235 | -0.085033593 | 1.70439517 | 0.701017394 | 0.786763076 |
| 1 | -0.624820486 | -0.92012672 | -1.046579085 | -0.194469362 | -0.053852452 | 0.012955636 | 0.406119584 | 0.425089635 | 0.736687246 | 0.116356918 |
| 0 | -0.276088472 | -0.609470433 | -1.588071685 | 0.306450396 | -0.215327011 | 1.263365248 | 0.098439143 | 0.231390915 | 0.193424169 | -0.691565994 |
| 0 | -0.412548889 | 0.090230557 | -0.114561763 | -0.49543139 | -0.76061984 | -0.352488202 | 0.668169492 | 0.588831106 | -0.6213292 | 0.625241287 |
| 0 | 0.15835524 | -0.605082094 | -0.009400846 | 0.507928459 | 1.584671169 | -0.428406981 | 0.583422173 | 0.104236116 | 1.781314108 | -0.805528181 |
| 1 | -0.223300522 | 0.125793403 | -1.473265316 | -0.931917688 | -0.220644817 | 1.272310276 | -3.769913968 | 0.104735102 | 0.540894384 | 1.494829398 |
| 1 | 1.712216099 | -0.737958656 | -0.191115652 | -0.468593173 | 1.363614643 | 2.075515859 | -0.690690175 | -0.928456334 | -0.108562274 | 0.115437872 |
| 0 | -0.069846244 | 0.614214633 | 0.496510599 | -0.505919486 | -1.073862233 | -0.169570614 | 0.855436411 | 0.320263444 | -0.903486865 | -0.203725972 |
| 0 | -0.491319609 | -0.510492828 | -0.760329257 | 0.343398001 | -0.818931912 | 0.113661395 | 0.151531449 | 0.06385723 | 0.130286088 | -1.061435538 |
| 0 | -1.547484913 | 0.675017294 | -1.182754048 | -0.040502313 | -0.780174496 | 0.456624511 | -1.084583344 | -1.005672006 | 0.456236488 | -0.401819848 |
| 1 | 0.902478357 | -0.056445067 | 1.726410163 | -0.53474063 | -0.358977891 | -0.385512865 | -0.102249729 | 0.116998879 | 0.150240853 | 0.147248549 |
| 0 | -1.057901702 | 0.848882808 | 0.805051342 | -0.132289434 | 0.249006963 | -0.356492823 | 0.833845855 | -0.114725769 | 1.588780018 | 0.656860987 |
| 1 | 0.849443668 | -0.715915021 | 0.2929128 | 0.67945094 | 0.165641199 | -0.413236369 | 1.054918318 | 1.28310131 | 0.090262661 | -1.024186143 |
| 1 | 0.69564209 | -0.299802237 | 0.429872226 | -0.442749772 | -0.071594482 | 0.774336249 | 0.064069226 | -0.254840518 | 0.19053847 | -0.368188554 |
| 1 | 0.662181666 | 0.204725201 | 0.487606318 | -0.828320574 | 1.078196273 | 0.274664032 | -0.510524673 | -0.617769768 | 0.108440079 | 0.899672069 |
| 1 | 2.506809682 | -1.030929748 | 0.193042912 | 1.417480169 | -0.165842407 | 1.758902456 | 0.364722005 | 2.197064027 | -0.664730184 | -1.418315509 |
| 1 | -0.510756208 | -0.833361173 | -0.942387511 | -0.583465434 | 0.170082951 | -0.435767255 | 0.392861898 | 0.040427903 | -0.197847081 | 0.890941379 |
| 1 | 1.488227966 | -0.669059577 | 0.162167107 | 0.720387642 | -0.87406028 | 0.499008087 | 0.676067259 | 0.418520615 | -2.257720332 | -1.18726541 |
| 1 | 0.583299515 | -0.638347152 | -0.036958972 | 0.554219175 | -0.644973894 | 0.124376622 | 0.524652519 | 0.460031976 | -0.68638807 | -1.060765075 |
| 0 | 0.179101722 | -0.602839354 | 0.895565645 | 0.059028385 | -0.906472064 | -0.422473808 | 1.141312066 | 0.001432647 | -0.204449837 | -0.547744609 |
| 0 | -0.698051074 | 1.111826007 | 1.174714798 | -0.504433792 | 0.99091548 | -0.803771659 | 0.386970717 | -0.663686283 | 1.006268104 | 1.482143368 |
| 1 | 0.427989106 | 0.42972632 | -0.305414477 | 0.441010125 | -0.699794874 | 1.316228269 | -0.717024824 | 1.156578961 | 0.376168146 | -0.92224518 |
| 1 | 0.186611133 | -0.877425359 | 0.253536064 | -0.284102683 | -1.335812519 | -0.476306698 | 0.842067595 | 0.867933112 | -0.648628716 | -0.169301976 |
| 0 | -0.72553045 | -0.169389184 | -0.945142569 | -0.170918052 | 0.02947905 | -0.478986292 | 0.012536706 | 0.628497005 | 0.415959746 | 0.208375492 |
| 1 | 0.669428172 | 0.821528013 | 2.692381392 | -0.274948184 | 0.658890423 | -0.593696688 | 0.772120499 | -0.116942564 | 1.852975001 | -0.530253731 |
| 0 | -0.980523896 | 0.399199486 | 0.123829922 | -0.03888146 | -0.746771067 | -0.412823882 | 0.139101439 | 0.122934411 | 0.614774793 | 0.098146618 |
| 1 | 0.655487318 | -0.469806428 | 0.293406713 | 0.957049456 | -0.017878495 | 0.119796792 | 0.63821573 | 0.478923929 | -0.009751533 | -1.083969856 |
| 0 | 1.031982081 | -0.50224427 | 2.179650465 | -0.476989733 | -0.533458148 | -0.230585149 | 0.596004454 | 0.10385814 | 0.61783653 | -0.136902054 |
| 1 | 0.615682265 | 0.645009559 | 0.352840898 | -0.12299715 | -0.314533163 | 0.384183558 | -0.881879856 | 0.661072896 | -0.113442132 | -0.298289787 |
| 1 | 0.57843411 | -0.575292345 | 0.256494574 | -0.304208383 | 0.140790191 | -0.773419074 | -0.097795605 | 0.269126252 | -0.008595813 | -0.540503571 |
| 1 | -0.624006401 | -0.447591072 | -0.980492174 | -0.820463088 | -0.305866433 | -0.49335535 | -1.542473939 | -0.430275169 | -0.878666893 | 1.880355674 |
| 1 | -0.205674943 | -0.121437615 | -1.370198127 | 0.975169728 | 0.798870262 | -0.479202498 | 0.187461945 | 2.300237792 | 1.236954031 | -1.021783741 |
| 0 | 0.679384248 | -1.065315216 | 0.998750453 | 0.070527157 | -0.854668604 | -0.582333017 | 0.920289865 | 0.121104517 | -0.711029319 | -0.271965327 |
| 0 | -0.491200979 | -0.225554035 | 0.766791474 | -0.337166519 | -0.219642605 | -0.394608556 | 1.065352448 | -0.205298879 | 1.071061696 | 0.043370959 |
| 1 | 0.449292331 | 0.389382883 | -0.196292916 | -0.297093453 | 0.887493314 | 1.418241578 | -0.576856028 | -1.410699404 | -0.055692123 | -0.013848143 |
| 1 | -0.142686829 | -0.179438652 | -0.240073965 | -0.344520661 | 0.194474478 | -0.325901485 | 0.47240375 | 0.352268946 | -0.03843054 | 0.462309126 |
| 1 | 0.883316463 | -0.174038368 | 2.179789253 | -0.255039529 | 1.001994562 | -0.512442143 | 0.68398416 | -0.03055625 | 1.275890469 | 0.800770548 |
| 0 | -0.706046553 | 0.592120316 | 2.719348814 | -0.931478494 | 0.139911575 | -0.72139903 | 1.040328162 | -0.310404452 | 1.554512242 | 3.360411047 |
| 0 | -0.751922999 | -1.114643384 | -1.764234398 | -0.133198242 | -1.773371751 | 0.729476177 | -0.340242906 | 0.145012073 | -1.336325718 | 0.564110465 |
| 1 | 0.94718641 | 0.369495022 | -1.380990813 | 0.555810111 | 1.49043477 | 0.637810178 | -1.415041777 | -2.113777426 | -2.688413772 | -0.574094165 |
| 1 | -0.655773119 | -0.376319239 | -0.956052886 | -0.63838983 | -0.239439649 | -0.306973569 | -0.703127223 | -0.798272329 | -0.228722896 | 0.078702754 |
| 1 | 3.234197439 | -0.855649576 | -0.091647426 | -0.122333551 | 2.16981168 | 0.691873781 | -1.964863391 | 1.397629924 | -0.353806836 | -0.468964123 |
| 0 | 0.565750073 | -1.047379638 | 0.387229656 | 0.259155619 | -0.487574782 | -0.048833536 | 0.54457771 | 0.021019321 | 0.005777872 | -0.955755696 |
| 1 | 0.857883071 | -0.942658403 | 0.499244463 | 0.612110883 | 0.164815736 | -0.6137329 | 0.473826929 | 0.64787134 | 0.108787881 | -0.872355977 |
| 1 | 0.215841345 | -0.206974143 | 0.143185986 | 0.38249774 | 0.483092882 | 0.152679356 | 0.832515269 | 0.232145622 | 0.774532421 | -0.811082932 |
| 1 | -1.170039862 | 0.596634111 | 0.108178972 | -0.795347343 | -1.60501235 | 1.047093008 | -1.201749806 | -1.724552582 | -0.256477034 | 1.26352565 |
| 0 | -1.514684054 | 1.836101956 | -1.487532225 | -0.07085454 | 0.024926512 | 0.798280602 | -1.016450116 | -2.651864642 | -1.264809855 | -0.306894406 |

1= Positive for LN Metastasis 0= Negative for LN Metastasis

Appendix E2 The distributions of the Radscore for each patient in the test cohorts

| label | Radscore | PLBP_hist_tumor_orient4_5 | original_shape_Maximum2DDiameterColumn | original_glcm_ClusterProminence | WL_lbp_hist_cV1_1 | WL_lbp_hist_cV2_1 | WL_lbp_hist_cV2_8 | WL_lbp_hist_cH2_7 | WL_lbp_hist_cH2_0 | original_glszm_GrayLevelNonUniformityNormalized |
| --- | --- | --- | --- | --- | --- | --- | --- | --- | --- | --- |
| 1 | 1.909783272 | -0.907732623 | -1.212378248 | 0.176441277 | 1.883842253 | 0.479169029 | -1.365616465 | 0.174635143 | -0.978343417 | -0.611726108 |
| 1 | -0.161929938 | -1.016533215 | 0.353854608 | -0.480617236 | -0.820650081 | 0.010003557 | 1.142641562 | -0.083115678 | -0.119953696 | 0.677802304 |
| 0 | -0.90187989 | -0.583782742 | -0.025750982 | -0.118134688 | -1.064921736 | -0.747523272 | 1.032575626 | -0.12887901 | -0.004218066 | -0.121096482 |
| 1 | -0.69281289 | 1.58752875 | -0.787081192 | 1.584429637 | 0.280785079 | 0.454808694 | -0.590951799 | -0.210693911 | -0.265480349 | -0.869813291 |
| 1 | 1.388340844 | -1.101593551 | -0.722966763 | 0.515667358 | -0.359361165 | 0.177291204 | -0.152636053 | 0.416980897 | -2.072419658 | -1.158389115 |
| 0 | -0.870115096 | 0.513899591 | -0.116003769 | 0.106312518 | -0.276584881 | -0.646269919 | 0.372926595 | 0.100739474 | -0.166903633 | 0.521576691 |
| 0 | -0.140047467 | 0.693283933 | -1.150894628 | -0.326913896 | -0.172450601 | 0.849863165 | -2.62670221 | -0.947462655 | -0.772570853 | 0.182075147 |
| 1 | 0.49192344 | 0.077251561 | 1.158796094 | 0.015401117 | -0.273807506 | -0.78980495 | 0.474074865 | 0.277902312 | 0.094639309 | -0.791958399 |
| 0 | 5.216229446 | -0.870138257 | -1.959157185 | 0.264609315 | 3.8226538 | 3.783698085 | -0.3488709 | 4.177220825 | -0.988908242 | -0.909225995 |
| 1 | -0.198140261 | -0.612977851 | 0.179740051 | -0.063385275 | 1.427071936 | -0.847436392 | 0.714434289 | -0.261894411 | 1.359661217 | 0.325562076 |
| 0 | -0.39926367 | -0.699751413 | -0.180725401 | -0.816808235 | -0.692486093 | 0.256127805 | 0.522175439 | 0.144360942 | 0.105446217 | 1.015493452 |
| 1 | -0.292753835 | 0.128939045 | 0.582397452 | -0.225920016 | -0.323799299 | 0.120600093 | 0.490927177 | -0.158346614 | 0.662934165 | 0.098996628 |
| 0 | 0.769864017 | -0.472004102 | 0.311781173 | -0.294512882 | -0.443437442 | -0.503395042 | 0.117760108 | 0.631042999 | -0.718085232 | -0.477326911 |
| 0 | -0.00290954 | 0.305346778 | 1.894873173 | -0.713503079 | 0.109363329 | -0.769189333 | 0.785399202 | 0.034229387 | 0.476778817 | 1.914221863 |
| 0 | -1.037618389 | 0.781043426 | 1.506618419 | -0.746042814 | -0.363311416 | -0.588824795 | 1.725188844 | -0.084394487 | 1.065953429 | 1.290088542 |
| 0 | -0.456399883 | -0.925943348 | -1.475457807 | -0.486081106 | -0.085652982 | 1.149006865 | -0.451117385 | 0.805509359 | 1.143193692 | 0.745131807 |
| 0 | 0.153958756 | 1.541757433 | -0.05820962 | -0.034168916 | -0.611334801 | -0.111986713 | -0.892034625 | 1.392814206 | -1.180303947 | 0.418218968 |
| 1 | 2.565077551 | 0.113029947 | -0.227731106 | 0.392059216 | 1.857184702 | -0.094397807 | -0.592452104 | 2.844086186 | -0.568821745 | -0.786238199 |
| 0 | -0.693703824 | 2.114014587 | 0.047206298 | 0.118945086 | -1.245230511 | 1.247570313 | -0.943943956 | -0.466758473 | -0.872812358 | 0.257097955 |
| 1 | 0.782435911 | -0.211430393 | -1.198134877 | 1.403258607 | 0.375435491 | 1.307706565 | -0.197535468 | 1.185038836 | -0.159778745 | -1.291793341 |
| 0 | -0.844637775 | -0.142364584 | -0.037352021 | -0.466363455 | -0.041161506 | -0.228612727 | 0.585828768 | -0.32696109 | 0.003554939 | 1.776243668 |
| 1 | 0.685975655 | -0.247873682 | 0.588905342 | 0.31786845 | 0.712662763 | 0.300797014 | 0.415696496 | -0.305907762 | 0.796406753 | -1.016919005 |
| 1 | 0.42032189 | -1.091391511 | -1.015435482 | 1.6393932 | -0.686843609 | 0.558083933 | 0.062851752 | -0.473240939 | -1.843223948 | -1.180443423 |
| 1 | -1.977209024 | 0.632267652 | -1.908166917 | -0.751409181 | -0.593642082 | -0.404220554 | -1.093198253 | -0.85558832 | -0.550018551 | 0.865822899 |
| 1 | 0.651755399 | -0.851812443 | 0.352677942 | 1.063425345 | -1.100075673 | -0.574066321 | 0.33758258 | 0.431416635 | -1.339262203 | -1.008017574 |
| 0 | -0.315150081 | 2.201167395 | -1.961230163 | -0.561610604 | -0.113191391 | 1.027829335 | 0.010265033 | 2.335454534 | -1.403228833 | 0.367717546 |
| 0 | -1.364625954 | 0.02905177 | -0.072263498 | -0.418241773 | -0.143088999 | 0.332424289 | 0.849358758 | -0.563415939 | 1.81223143 | 0.309848924 |
| 0 | -2.191774749 | 3.164110583 | 0.839560228 | -0.19146538 | -0.493268498 | -1.078713376 | 0.648747607 | 0.26582057 | 1.234914826 | 0.141853863 |
| 1 | 0.556601414 | 2.689823793 | 3.721722227 | -0.844047312 | -0.397965528 | -0.340887876 | -0.036364679 | 0.218737954 | 0.161438901 | 1.895214868 |
| 0 | -0.45846763 | -0.859129109 | -0.140513973 | -0.657308804 | -1.484063326 | -1.547191604 | -0.049598473 | 0.603050146 | -0.991766474 | 0.517799849 |
| 1 | 0.193627279 | -0.198309721 | 0.819736495 | 0.165482901 | -0.823727479 | -0.466028428 | 0.128780422 | 0.515095885 | 0.174488764 | -0.413441072 |
| 1 | 0.790490021 | -1.062439874 | 0.570153243 | 0.732954392 | 0.308169154 | 0.138910326 | 0.991382464 | -0.191842848 | 0.222426075 | -0.870400505 |
| 0 | 0.32691263 | -0.307233464 | 3.542334551 | -0.653189656 | 0.155969696 | -0.44610198 | 1.008097176 | -0.330710131 | 2.037876478 | 2.574653547 |
| 0 | -0.869054867 | 0.976425318 | -1.06117816 | 0.300599491 | 0.018078812 | 0.75387514 | 0.080557713 | 0.166540104 | 0.738025841 | -1.009080806 |
| 1 | 0.544829579 | -0.387787157 | 1.15974798 | 1.728517535 | 0.666922845 | -0.909626691 | 0.186272031 | -0.409239068 | 0.457445923 | -1.034952336 |
| 1 | 0.558305984 | 0.651341014 | -0.199912399 | -0.348314213 | -1.30204791 | -0.82666382 | -0.966936872 | 1.898176085 | -1.731816013 | -0.281397028 |
| 1 | 0.152286747 | -0.312756708 | -0.52691663 | -0.441791711 | -0.393576929 | -1.010199965 | -1.213314568 | 0.321754385 | -0.951210661 | 0.039633905 |
| 0 | -1.562622749 | -0.923804131 | -0.315437937 | -0.257764336 | -1.185236452 | -1.067061314 | 1.313180287 | -0.284348389 | 0.444555861 | 0.14217698 |
| 0 | 0.97653066 | -0.90696463 | 0.349341724 | 0.355207519 | -0.572160491 | 0.253384766 | 0.061233576 | -0.786735238 | -1.408438426 | -0.763427898 |
| 0 | -0.948204444 | 1.242112837 | -1.505423599 | -0.626473568 | -1.350447051 | 2.587456771 | -1.603121602 | -0.406537401 | -0.150989557 | 0.371989912 |
| 1 | 0.65164439 | -1.025764009 | 0.008791145 | 1.394448617 | -0.912633904 | -0.527618174 | -0.433772274 | -0.559899579 | -1.679196734 | -1.385611794 |
| 1 | 3.663303412 | 1.918164422 | -0.369880709 | -0.524606319 | 5.876813512 | 6.345461408 | 2.270464238 | -3.553521655 | -1.708536065 | 0.133515212 |
| 1 | -0.223159711 | -0.663597622 | 0.312174119 | 0.245308618 | -0.704255837 | -0.183758773 | 0.993984057 | -0.247497695 | 0.257083161 | -0.803851179 |
| 0 | 0.41014173 | -0.777487691 | -0.586415558 | -0.856119273 | -0.093985748 | 0.545098978 | -0.683000788 | 0.755743756 | -0.058212075 | 1.151962488 |
| 1 | 0.450236326 | -0.702203548 | 0.449055738 | 0.351550262 | -0.413766532 | -0.378752621 | -0.081776223 | 0.03127449 | -0.051446831 | -0.672919851 |
| 1 | 0.458762908 | -0.101856817 | 0.354984064 | -0.24337788 | -0.111844649 | -0.493411805 | -0.23583915 | 0.399425455 | -0.030965297 | -0.426983249 |
| 0 | 0.213154478 | -0.359308112 | 0.371026193 | -0.529751187 | -0.515327081 | ################### | -0.43548106 | -0.510437229 | -0.409596625 | 0.398592636 |
| 0 | -0.89595954 | 3.54366259 | -1.495267613 | -0.588533399 | -0.26711414 | 2.090476208 | -1.422969285 | 0.125871042 | -0.977282552 | 0.162223776 |
| 1 | 1.231159204 | -0.979412449 | -0.190817146 | -0.279619393 | -0.408104196 | -0.222344845 | 0.795601654 | 0.753964716 | -1.893264678 | -0.455936884 |
| 1 | 0.876608807 | -0.836448839 | -0.369033999 | 1.778853869 | 0.415696545 | 0.860665608 | -0.08856051 | 1.211157583 | 0.891390444 | -1.380846162 |
| 0 | -0.038271206 | -0.493643199 | -0.639704697 | -0.400551053 | -0.217328233 | 0.135342487 | -0.031637042 | -0.239221036 | -0.400316679 | -0.145589557 |
| 1 | 0.380380908 | -0.921206288 | 0.64423096 | 0.805725526 | -0.924574633 | -0.49621864 | 0.907943129 | -0.521087602 | -0.939863554 | -1.151908862 |
| 0 | -1.842401659 | 2.967146823 | -0.746249891 | -0.617284994 | -1.009566989 | -0.749855398 | -0.548262197 | 0.270224479 | -0.96364842 | 0.111846258 |
| 1 | 0.118688468 | -0.964976513 | 0.804125832 | -0.018771681 | -0.156276889 | -0.452996457 | 1.045918667 | 0.064354971 | 0.960700465 | -0.395125621 |
| 0 | 0.610605882 | 0.731870997 | 0.111430665 | -0.477547878 | 1.122840483 | 0.815063234 | -0.157382526 | -0.105485178 | 0.247533177 | 0.244815896 |
| 0 | -0.722631644 | 0.121156553 | 1.151002124 | -0.891152571 | -0.274697969 | -0.547054881 | 1.601619864 | 0.329099906 | 0.155588028 | 2.735358223 |
| 0 | 1.52467961 | 0.184340031 | 3.121524165 | 1.220885547 | 0.437000428 | -0.43204718 | 0.693808215 | -0.145351585 | 1.011546671 | -1.161856323 |
| 1 | -0.335576288 | -0.879663827 | -1.237024156 | -0.722723638 | -0.114803255 | 0.263762487 | -0.481578914 | 0.278603467 | -0.027789049 | 0.976373112 |
| 1 | 0.621375012 | 0.301919126 | 0.154200626 | -0.411884617 | -0.548494846 | -0.396813614 | -1.59222481 | 0.201537656 | -1.145926421 | 0.112049396 |
| 0 | 0.880161591 | -0.918974011 | 0.343411766 | 0.947809276 | -0.489866101 | 0.947319714 | 1.237928887 | 0.091258315 | -0.366958024 | -1.264006446 |
| 1 | 1.718250766 | 1.52292951 | -1.017093026 | -0.588209146 | -0.343893484 | 1.393877047 | -0.88873433 | 5.547998434 | -0.289573952 | 0.011441489 |
| 0 | 0.953719174 | 0.080502837 | 0.273678226 | -0.504351061 | 0.22945551 | -0.486661698 | -0.15285177 | 0.392091142 | -1.057484814 | -0.119177673 |
| 1 | 0.763796234 | -0.915190294 | 0.575911262 | -0.416367133 | -0.472191633 | -0.275368142 | 0.425234745 | 0.413074729 | -0.778344816 | 0.556765532 |
| 0 | -0.745914206 | -0.338331182 | -1.309370042 | -0.888026087 | 0.79767177 | -0.098266098 | 0.051777906 | -0.290900389 | -0.752947166 | 2.559032747 |
| 0 | -0.742059116 | 0.293550824 | -0.449744519 | -0.610114472 | 0.197074465 | -1.433434298 | -1.16663737 | 0.005573778 | 0.32950607 | 0.350236957 |
| 1 | 0.397112031 | 0.089509885 | -1.41511396 | 1.054427616 | 1.132753413 | 0.351103139 | -1.941676171 | -0.264746563 | -0.413897096 | -0.636356752 |
| 0 | -0.315263376 | -0.210686877 | -0.391473849 | 0.148115135 | 0.08056175 | -0.240538848 | 0.273029901 | 0.577860667 | 1.03497995 | -0.855573525 |
| 0 | -0.555464146 | -0.5465466 | -0.600705766 | 0.249328348 | -0.434508472 | -0.569437901 | 0.608852614 | -0.48178079 | -0.592093392 | -0.537291768 |
| 1 | 0.044084066 | -0.147637493 | -0.97034508 | 0.707027421 | 0.348936757 | -0.394831928 | -1.295801912 | -0.282727513 | -0.496587692 | -0.777005347 |
| 1 | -0.692746147 | -0.486560117 | 0.105501476 | -0.269041638 | -0.614172373 | -0.209877597 | 0.69091695 | -0.114822701 | 0.908643359 | -0.199599663 |
| 1 | -0.712529774 | 0.905553991 | 1.784820155 | -0.571695273 | -0.397156394 | -0.60737955 | 0.857964149 | -0.253095726 | 1.627207143 | 0.155049903 |
| 1 | 1.980763854 | -1.082022591 | -0.118501087 | 1.46468982 | -0.02481713 | 0.361859091 | 0.216596357 | 1.302239228 | -1.627202499 | -1.39107652 |
| 0 | -5.982651741 | -0.614450293 | -0.229105121 | -0.909786835 | -2.082554386 | -1.461304444 | -2.339848095 | -6.451084675 | 3.835781953 | 1.983002758 |
| 0 | -0.561464499 | 1.248556117 | 0.024583119 | -0.5902981 | -0.112127061 | 0.231553535 | -0.647987388 | -0.181057557 | -0.650335867 | -0.089954785 |
| 1 | -0.126238459 | 0.129319298 | 0.024438276 | 0.175830193 | 1.558053642 | -0.484588601 | 0.548527635 | -0.085356551 | 1.581414269 | -0.86903348 |
| 1 | 0.411529758 | -0.429942399 | -0.923570464 | -0.217145354 | -0.755975405 | -0.172494029 | 0.235610401 | -0.008516364 | 0.012004504 | -0.125291348 |
| 0 | 0.159376317 | -0.829741211 | -1.099362408 | 0.20291668 | -0.470534301 | -0.65994544 | 0.515818226 | -0.089144822 | 0.498133215 | -0.975247082 |

1= Positive for LN Metastasis 0= Negative for LN Metastasis
